# Representing virus-host interactions and other multi-organism processes in the Gene Ontology

**DOI:** 10.1186/s12866-015-0481-x

**Published:** 2015-07-28

**Authors:** R. E. Foulger, D. Osumi-Sutherland, B. K. McIntosh, C. Hulo, P. Masson, S. Poux, P. Le Mercier, J. Lomax

**Affiliations:** European Molecular Biology Laboratory, European Bioinformatics Institute (EMBL-EBI), Wellcome Trust Genome Campus, Hinxton, Cambridge CB10 1SD UK; Department of Biochemistry and Biophysics, Texas Agrilife Research, Texas A&M University, College Station, TX 77843 USA; Swiss-Prot Group, SIB Swiss Institute of Bioinformatics, Centre Medical Universitaire, 1 Rue Michel-Servet, 1211 Geneva 4, Switzerland

**Keywords:** Annotation, Gene Ontology, Host, Ontology, Virus

## Abstract

**Background:**

The Gene Ontology project is a collaborative effort to provide descriptions of gene products in a consistent and computable language, and in a species-independent manner. The Gene Ontology is designed to be applicable to all organisms but up to now has been largely under-utilized for prokaryotes and viruses, in part because of a lack of appropriate ontology terms.

**Methods:**

To address this issue, we have developed a set of Gene Ontology classes that are applicable to microbes and their hosts, improving both coverage and quality in this area of the Gene Ontology. Describing microbial and viral gene products brings with it the additional challenge of capturing both the host and the microbe. Recognising this, we have worked closely with annotation groups to test and optimize the GO classes, and we describe here a set of annotation guidelines that allow the controlled description of two interacting organisms.

**Conclusions:**

Building on the microbial resources already in existence such as ViralZone, UniProtKB keywords and MeGO, this project provides an integrated ontology to describe interactions between microbial species and their hosts, with mappings to the external resources above. Housing this information within the freely-accessible Gene Ontology project allows the classes and annotation structure to be utilized by a large community of biologists and users.

## Background

The Gene Ontology (GO) is a bioinformatics resource to describe functional attributes of gene products across all kingdoms of life. The GO project is a collaborative effort developing three ontologies to describe the molecular actions of a gene product, the biological process those actions are part of, and the cellular locations in which they are active. First developed in 1998 [1], the Gene Ontologies arose from the need for standard descriptions to define a given object or process, and contained terms summarising the biology of three organisms (mouse, fly and yeast). GO has since grown to be the most popular bio-ontology used for describing gene product characteristics [2, 3]. It now contains more than 40,000 terms, expressed using the W3C standard ontology language OWL2 [4].

Subsequent to or in parallel with ontology construction, GO classes are associated with a gene product; this association is termed an annotation. Annotations are the result of manual analysis by trained curators and/or computational methods. Annotations are linked to an underlying source and include an evidence code [5] indicating the supporting data. Biological ontologies and related annotations have proven invaluable in interpreting accumulated biological data, where the volume of information and variations in terminology can pose problems. GO annotation in particular has been used in analyses ranging from small-scale queries about a protein or pathway of interest to large-scale high-throughput studies including, among others, gene enrichment analyses, microarray analyses, predicting gene functions and text-mining (e.g. [6–9] provide a representative overview). These analyses have allowed biologists to rapidly gain knowledge about their gene product or gene product set.

### The need for a microbe-and virus project

**Fig. 1:**
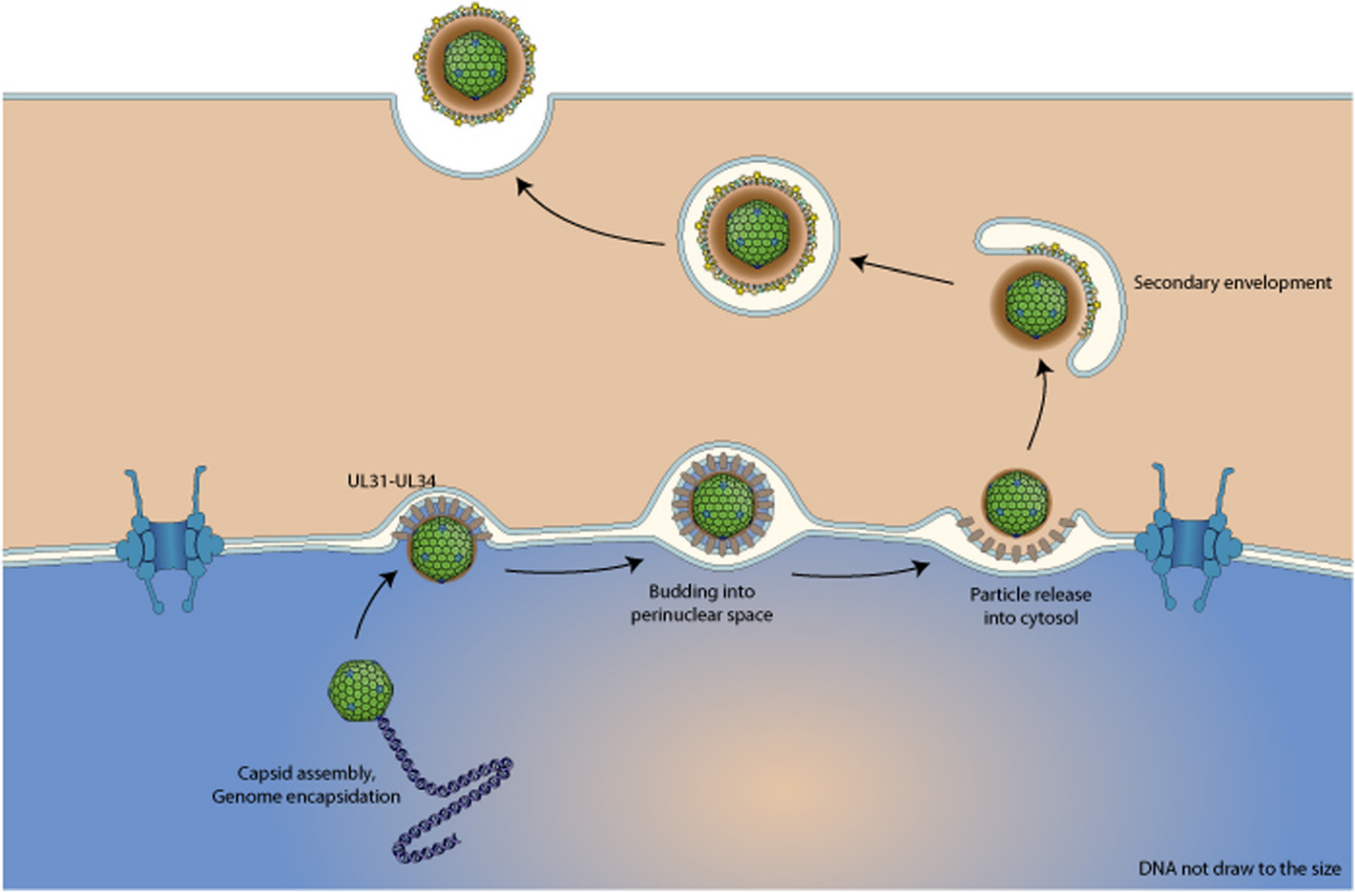
Nuclear egress represented in ViralZone and the Gene Ontology. Viral capsids assembled in the nucleus can migrate to the cytoplasm by utilizing an unusual export pathway termed ‘nuclear egress’. In this process, the viral capsid is delivered into the perinuclear space, producing a vesicular intermediate after fission. After fusion with the outer nuclear membrane, the naked capsid is released into the cytosol. The corresponding GO term ‘exit of virus from host cell nucleus by nuclear egress’ (GO:0046802) is mapped to ViralZone entry 1952. The sub-processes are described by GO terms ‘nuclear capsid assembly’ (GO:0039708) corresponding to ViralZone entry 1516, ‘viral budding from nuclear membrane’ (GO:0046765), ‘fusion of viral membrane with host outer nuclear membrane’ (GO:0039700) and ‘viral capsid secondary envelopment’ (GO:0046745)

Although GO was initially developed for eukaryotic organisms, primarily to support the work of eukaryotic model organism databases, GO has long been applicable to organisms beyond this taxonomic group [10, 11]. Despite this, GO remains largely under-utilized for prokaryotes, single-celled eukaryotic species, and viruses, as shown by manual annotation counts to these groups [12]. This project aims to promote microbial GO annotations, firstly by developing GO terms applicable to microbial processes and structures, and secondly by putting in place an annotation structure that allows description of both the microbe and the host environment. A number of microbial resources are already in existence; the integrated microbial genome network (IMG) [13] provides users with a set of tools to compare the genes, genomes and functions of microbial genomes. The MeGO vocabulary uses the GO format to describe the functions of mobile genetic elements, and the MeGO terms are used to annotate phage and plasmid protein families in the ACLAME database [14]. The Infectious Disease Ontology (IDO) and its sub-domain-specific extensions represent entities relevant to infectious diseases [15]. For viruses, many connected resources are available for data retrieval and analysis. ViralZone [16] provides molecular information for all virus groups, along with virion and genome figures in the form of fact sheets. The ViralZone pages have supplied information sources for many of the new viral GO terms (Fig. 1), and we have worked alongside the ViralZone team [17] to provide reciprocal mappings to both ViralZone pages and UniProtKB keywords (labels that can be used to retrieve particular subsets of UniProtKB entries) [18]. The Virus Pathogen Database and Analysis Resource (ViPR) [19] provides an integrated repository of data and analysis tools for human pathogenic viruses. The Virus Variation Resource [20] is a web-based resource initially designed for accessing large influenza sequence datasets [21], and now housing sequence data for multiple viral and viroid genomes including Dengue virus [22] and West Nile Virus, together with a set of displays to view and explore the viral sequence datasets. The VIPERdb database [23] describes icosahedral virus capsid structures from the Protein Data Bank (PDB) including detailed structural and computational analyses. The Virus (re-)annotation database ViRAD [24] annotates protein-coding sequences in RNA viruses represented in the NCBI RefSeq database. Alignments of the resulting sequence are analysed to identify novel protein-coding reading frames and non-coding functional elements embedded within the protein-coding regions.

### History of multi-organism process terms in GO

Early iterations of the GO contained very few classes to describe processes involving hosts and their symbionts. This was largely because little annotation had been generated for the species typically participating in these processes. The exception was *Plasmodium falciparum,* which was fully annotated with GO classes in 2002 [25]. The host/parasite classes that existed at the time had no common root class, and it was often ambiguous as to whether the process involved a second organism or not. For example, the process of cell lysis can either be induced in a host organism by its parasite, or can be an endogenous process whereby the immune system destroys its own infected cells. GO had only a single term for this process, which did not allow annotators to distinguish between endogenously and exogenously induced cytolysis.

In 2004 the PAMGO (Plant-Associated Microbe Gene Ontology) Consortium [26] worked with GO to develop a core set of terms for describing host-parasite interactions, specifically for the annotation of plant parasites [10, 27]. This branch, comprising 450 classes, subsumed the existing classes and formed a single branch in the biological process ontology with the root class ‘interaction between organisms’ (later renamed ‘multi-organism process’). It included host-parasite interactions involving macroparasites and microbial parasites as well as intra-species multi-organism processes such as biofilm formation.

Processes involving viruses were also sparse in early versions of GO. A set of classes was added in 2002 to accommodate a protein annotation set for Herpes simplex virus 1 (HSV-1), but not all of these classes were well suited for the annotation of other viral species, and the classes themselves were not well integrated with the rest of the biological process ontology. In 2009 we embarked on a project to improve the representation of viral processes in GO. This involved input from experts for a variety of viral species, including bacteriophages. We now report on the conclusion of these changes, including annotations for several viral species using the refactored terms.

With this project, we provide an integrated, comprehensive ontology to describe multi-species interactions and microbial biology, with mappings to relevant external resources, where possible. The advantage of housing these data in GO is that the Gene Ontology is a well-known and respected, sustainable, open-source resource, for which a battery of annotation and analysis tools have been built. Thus microbial annotations can reach a wide audience for analysis, and microbial researchers have a wide range of tools and services available for analysing and interpreting their data.

## Construction and content

### Ontology design challenges

Constructing and using GO terms for inter-species interactions presented a number of challenges and resulting ontology design decisions, which we describe below.

#### Diversity between organisms, and what constitutes an organism?

GO terms have to capture the diversity of biology seen across many species, including the great diversity seen within microbes themselves. Within the viruses alone, the genetic material can be DNA or RNA, double-stranded or single-stranded, and viruses come in all shapes and sizes. But it is not only the make-up of microbial organisms that exhibits diversity; they also vary in their biochemistry and replication mechanisms [28]. The classes we created had to encompass this diversity whilst remaining general enough to be used to make inferences across species. For example, different microbes use a variety of mechanisms to adhere to their host organisms: via type IV pili in the case of bacteria, via specific infective structures such as the appressorium in parasitic fungi and via receptor-mediated binding in viruses. These disparate processes have a generic grouping under the GO multi-organism process sub-tree of ‘adhesion of symbiont to host’ (GO:0044406).

We also had to decide whether viral processes belonged in the ‘multi-organism process’ sub-tree, and this hinges on whether viruses are ‘living’ organisms or not. This is a question that has prompted much discussion e.g. [29] and the research community is split on this issue. Ultimately we decided that for the purposes of this project, viruses would be deemed to be organisms, and as such viral processes would be subtypes of multi-organism processes. We reached this decision to align GO with other bio-ontologies that have modelled viruses; for example, the Infectious Disease Ontology (IDO) [15], Experimental Factor Ontology (EFO) [30] and Ontology for Biomedical Investigation (OBI) [31] explicitly state that viruses [32] are a subclass of organism [33].

#### Symbiosis and mutualism

One of the key terms we created in this project was ‘symbiosis, encompassing mutualism through parasitism’ (GO:0044403). Here, the usage of symbiosis is not synonymous with mutualism (an interaction from which both organisms benefit). Instead it is used in its broad sense, to mean *any* intimate association between two organisms of different species, regardless of whether the outcome of the interaction is beneficial to both species (mutualism) or detrimental to one species (parasitism).

#### Upper-level multi-organism processes

**Fig. 2:**
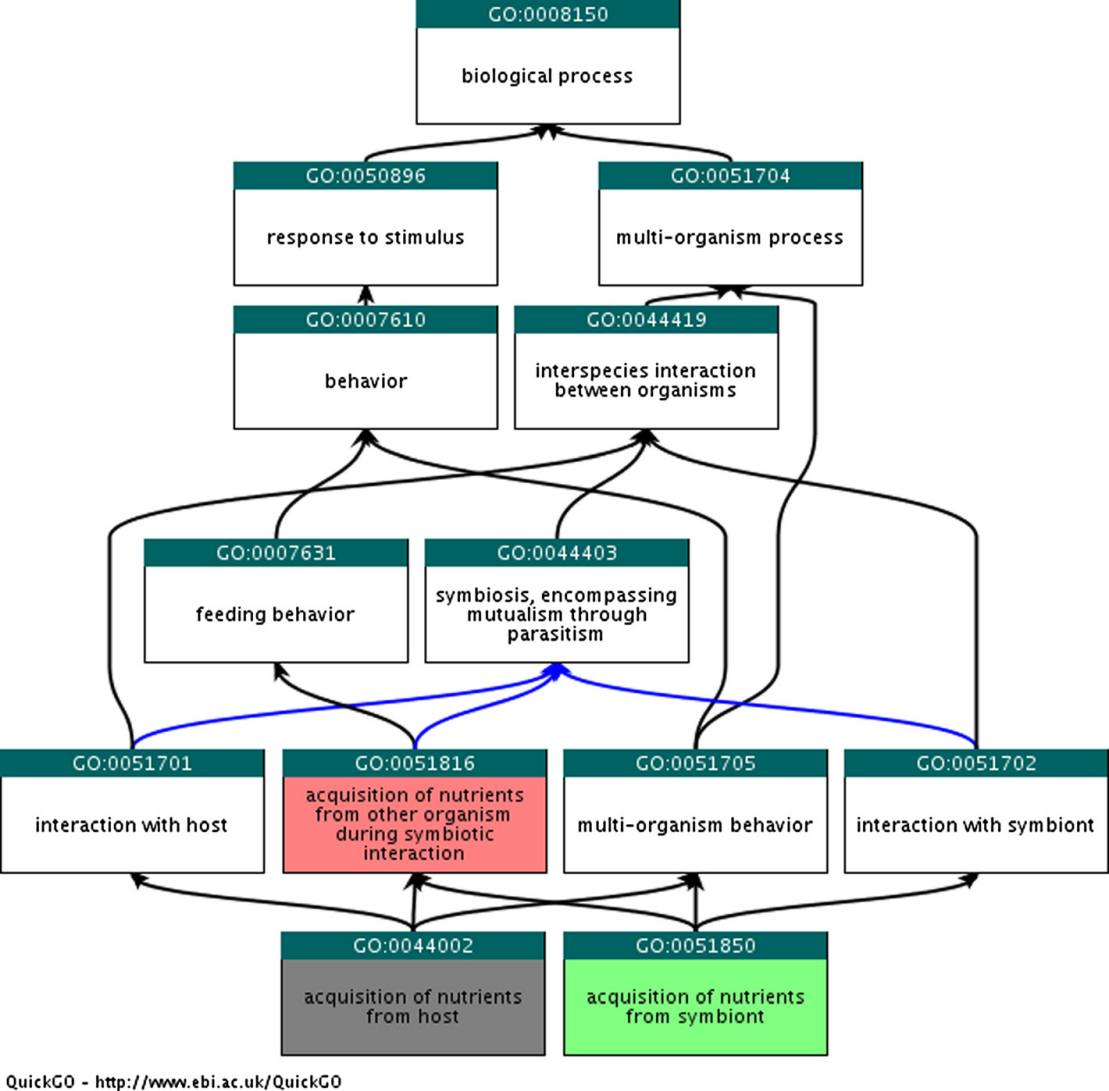
The triad structure in the multi-organism process node. The design of the upper-level classes in the multi-organism process subtree loosely follows a common ‘triad’ pattern in which there is a general parent class with two subclasses each specific for either the host or symbiont perspective. For example, ‘acquisition of nutrients from other organism during symbiotic interaction’ (GO:0051816) has the subtypes ‘acquisition of nutrients from host’ (GO:0044002) and ‘acquisition of nutrients from symbiont’ (GO:0051850). Full triads are only created where both subclasses are relevant. Blue arrows denote *part_of* relationships between classes, and black arrows denote *is_a* relationships between classes. The Gene Ontology uses multiple axes of classification, reflecting the multiple, scientifically valid ways that biological entities can classified. Thus any GO class can have multiple *is_a* parents. Image taken from QuickGO [12]

The upper-level classes in the multi-organism process subtree were designed to loosely follow a common ‘triad’ pattern in which there is a general parent class with two subclasses; one from the perspective of the host and the other from the perspective of the symbiont. For example, ‘acquisition of nutrients from other organism during symbiotic interaction’ (GO:0051816) has the subtypes ‘acquisition of nutrients from host’ (GO:0044002) and ‘acquisition of nutrients from symbiont’ (GO:0051850) (Fig. 2). Some symbiotic processes are only relevant to one of the participants, e.g. ‘dissemination or transmission of symbiont from host’ (GO:0044007) so the full triads are only created where a process is common to both host and symbiont.

#### Viral processes

**Fig. 3:**
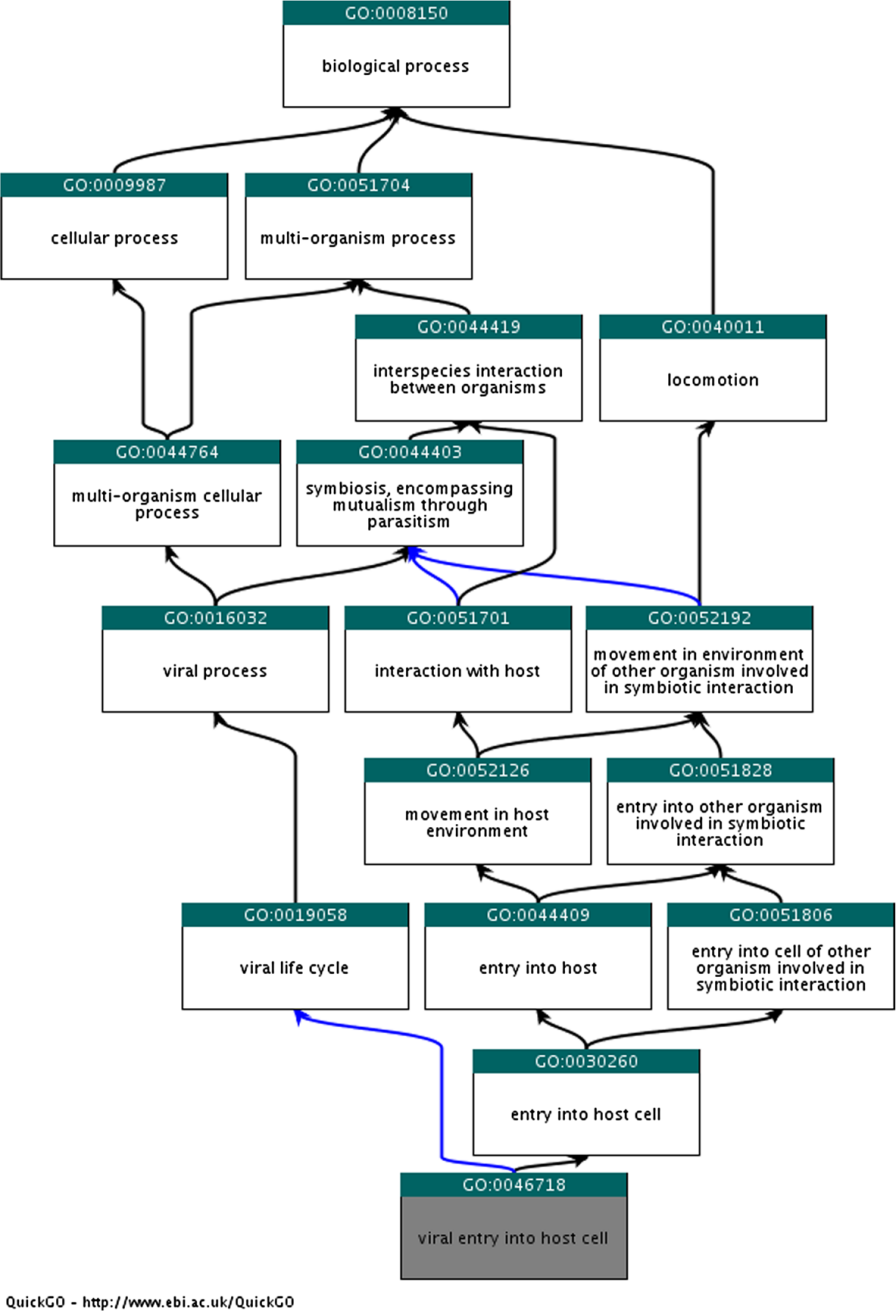
Placement of ‘viral entry into to host cell’ (GO:0046718). ‘Viral entry into host cell’ (GO:0046718) is placed in the ‘multi-organism process’ node, and can be used to annotate both host and virus gene products. Blue arrows denote *part_of* relationships between classes, and black arrows denote *is_a* relationships between classes. Image taken from QuickGO [12]

For viruses a slightly different top-level approach was required. This was because viruses rely largely on the host physiology and machinery to make copies of themselves and their parts, meaning there is a large overlap between viral and host processes. This made it difficult to delineate separate classes for host and viral perspectives. For example, during most viral transcription it is the host polymerase that provides the activity, so having a separate term describing only the virus contribution is not appropriate. We thus decided that viral processes should be largely agnostic as to whether the process is from the perspective of the host or the virus. For example, ‘viral entry into host cell’ (GO:0046718) can be used to annotate both the viral and host proteins involved in the entry process (Fig. 3). The annotation model is described in further detail below.

**Fig. 4:**
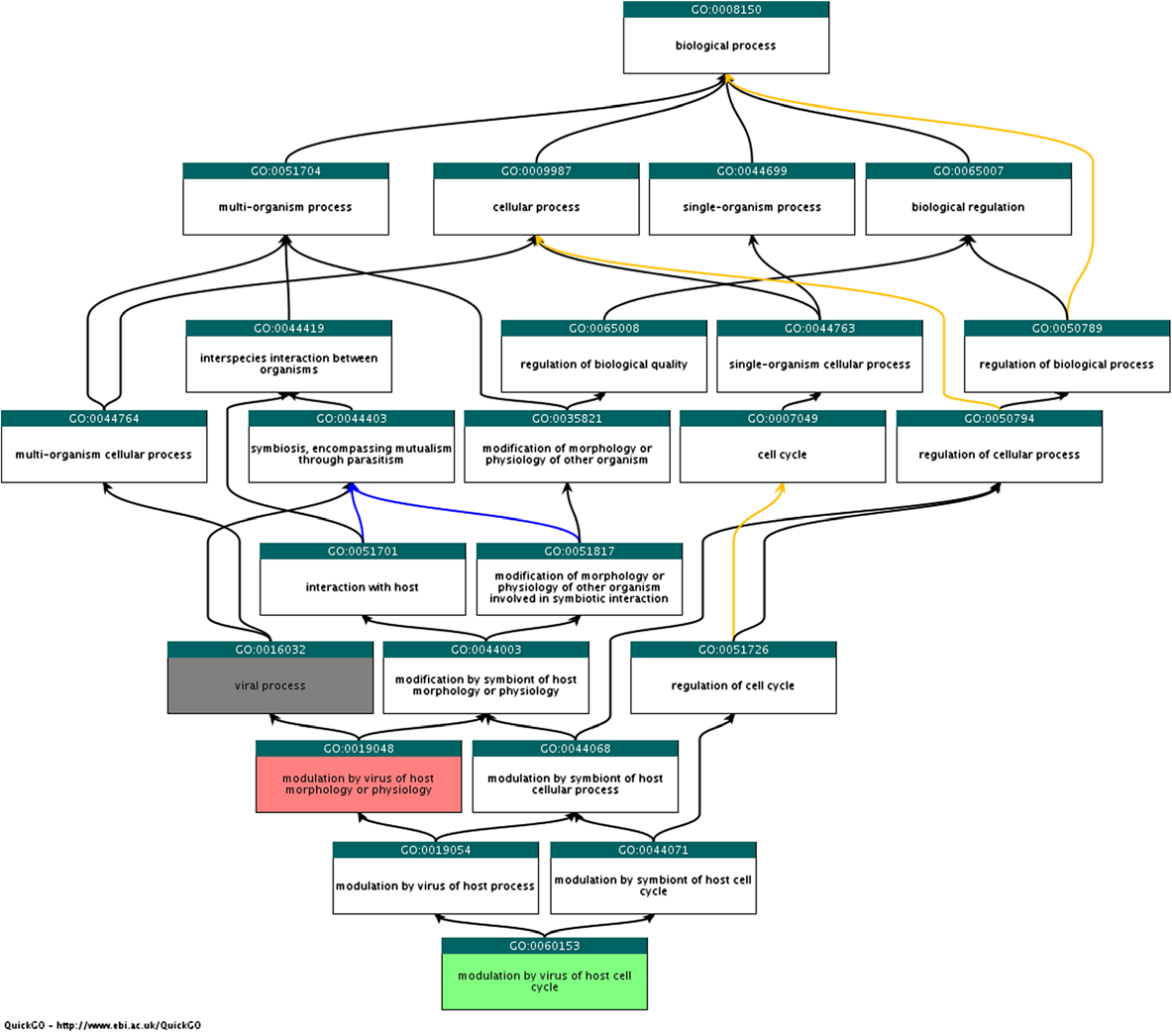
Placement of ‘modulation by virus of host cell cycle’ (GO:0060153). GO:0060153 is placed under ‘viral process’, and is connected to the single organism process term ‘cell cycle’ (GO:0007049) since ‘modulation by symbiont of host cell cycle’ (GO:0044071) *is_a* ‘regulation of cell cycle’ (GO:0051726). Blue arrows denote *part_of* relationships between classes, black arrows denote *is_a* relationships between classes, and yellow arrows denote *regulates* relationships between classes. Image taken from QuickGO [12]

We arranged viral processes under the top-level class ‘viral process’ (GO:0016032) which itself has two major subclasses, ‘viral life-cycle’ (GO:0019058) and ‘modulation by virus of host morphology or physiology’ (GO:0019048). This loosely mirrors the top-level divisions made in ViralZone [17], and allows us to capture the canonical host process that the virus is subverting or modifying (Fig. 4). We decided that phage processes were not distinct enough to require their own subtree, although some sub-processes will be unique to phages. For example the classes ‘viral genome ejection through host cell envelope’ (GO:0039678) and ‘viral DNA genome packaging, headful’ (GO:0098006) would only be applicable to phages and their hosts, with the phage-specific nature of these terms captured using synonyms. This is consistent with the way GO is modelled for single-organism processes that are specific to a species group. For the purpose of GO, we also decided that viruses do not ‘develop’ in the sense that multicellular organisms do. Thus new viral-specific classes are not subclasses or parts of the current ‘developmental process’ (GO:0032502) GO class.

### Automating ontology development for multi-organism processes

The initial set of microbial terms required great manual input. In order for the multi-organism node of GO to be sustainable, new GO terms should be added, at least in-part, automatically. To this end we are developing a set of design patterns for multi-organism processes that include logical definitions [34]. These will be used to construct templates [35] through which any registered user can request new terms which then need only minimal review by editors.

For example, we can make multi-organism specific subclasses of existing GO classes by specifying that the process must have multiple organisms as participants. For example: ‘multi-organism membrane fusion’ (GO:0044800) can be defined as any ‘membrane fusion’ (GO:0061025) in which there is more than one participating organism.^1^ Other logical definitions will take advantage of formally defined relations for recording relationships that hold between interacting organisms such as ‘*host_of’* , ‘*symbiont_of’* and ‘*vector_for’* , developed in collaboration with the Population and Community Ontology (PCO) [36].

### Virus GO-slim

**Fig. 5:**
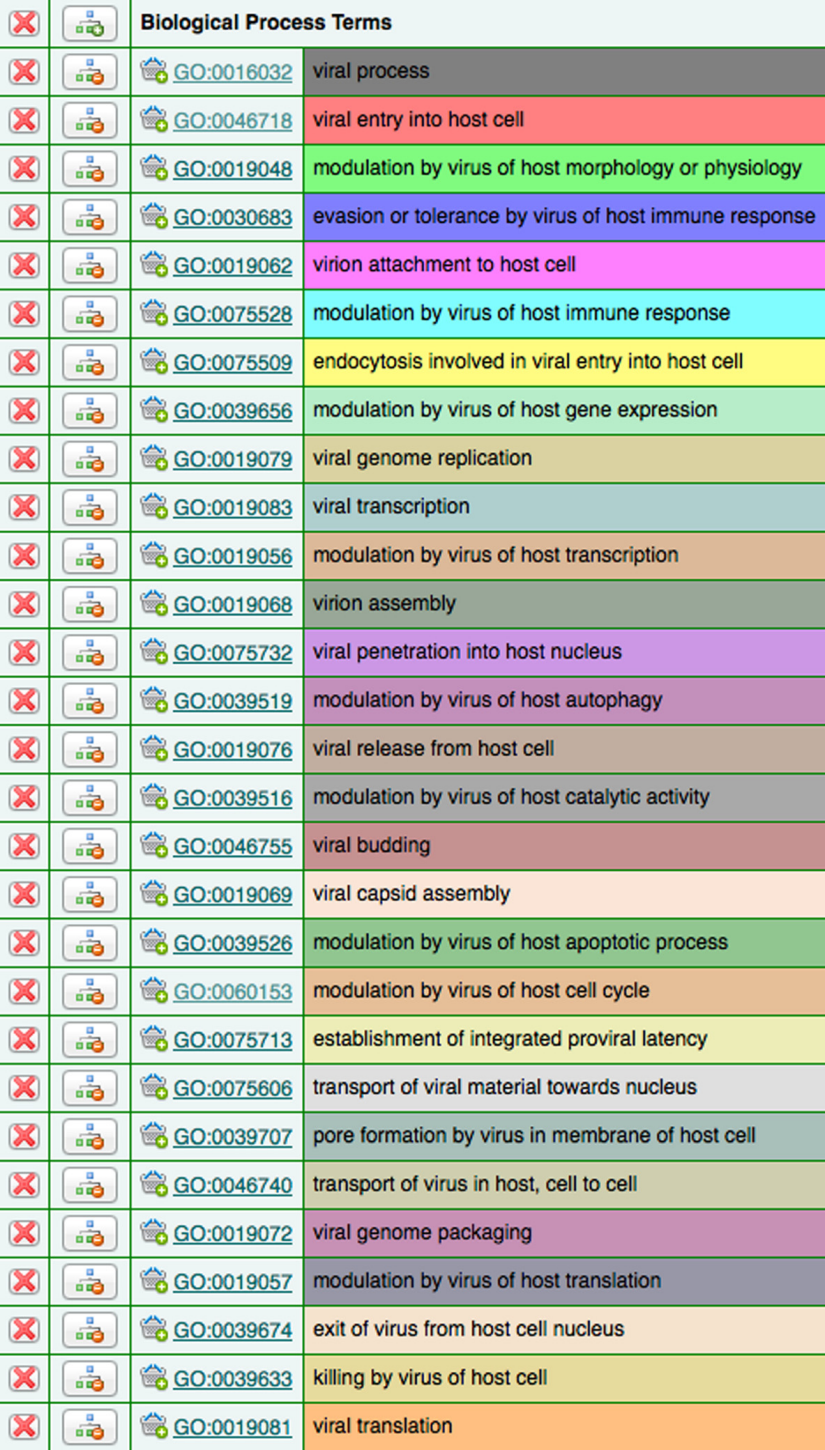
The viral GO slim contains 29 terms. The virus GO slim is a cut-down version of the Gene Ontology, containing 29 process terms which give a broad classification of viral processes. Image taken from QuickGO [12]

GO slims are cut-down versions of the Gene Ontology, containing a subset of the terms in the whole GO. They give a broad overview of the ontology content without the detail of the specific fine-grained terms, and can be created for specific areas of the GO, or for specific species. GO slims are particularly useful for giving a summary of GO annotation when broad classification of gene product process is required. We present a GO slim tailored to GO process terms suitable for annotation of viruses (Fig. 5). This provides the basis for a streamlined, accurate analysis of viral annotations. This is particularly pertinent for viral classes since many of them are only classified in the multi-organism branch, and therefore will not map to the broader terms present in the generic GO slim. In total there are 29 process terms in the viral GO slim, which is available from the GO website [37].

### Summary of ontology changes

As a result of this project, the multi-organism process node of GO has been improved and extended to over 2000 classes under ‘multi-organism process’ (GO:0051704) (2241 as of October 8th 2014). The collaboration with ViralZone and the focus on creating terms suitable for annotation of viral gene products has generated 344 classes under ‘viral process’ (GO:0016032) and 65 classes of ‘virion part’ (GO:0044423) in the component ontology. The objective of this project was to produce a collaborative resource, and towards this goal we have linked multi-organism GO terms to a number of external resources with 126 multi-organism terms now mapped to UniProtKB keywords and 148 mapped to ViralZone pages, providing comprehensive information for users.

### Annotation model

**Fig. 6:**
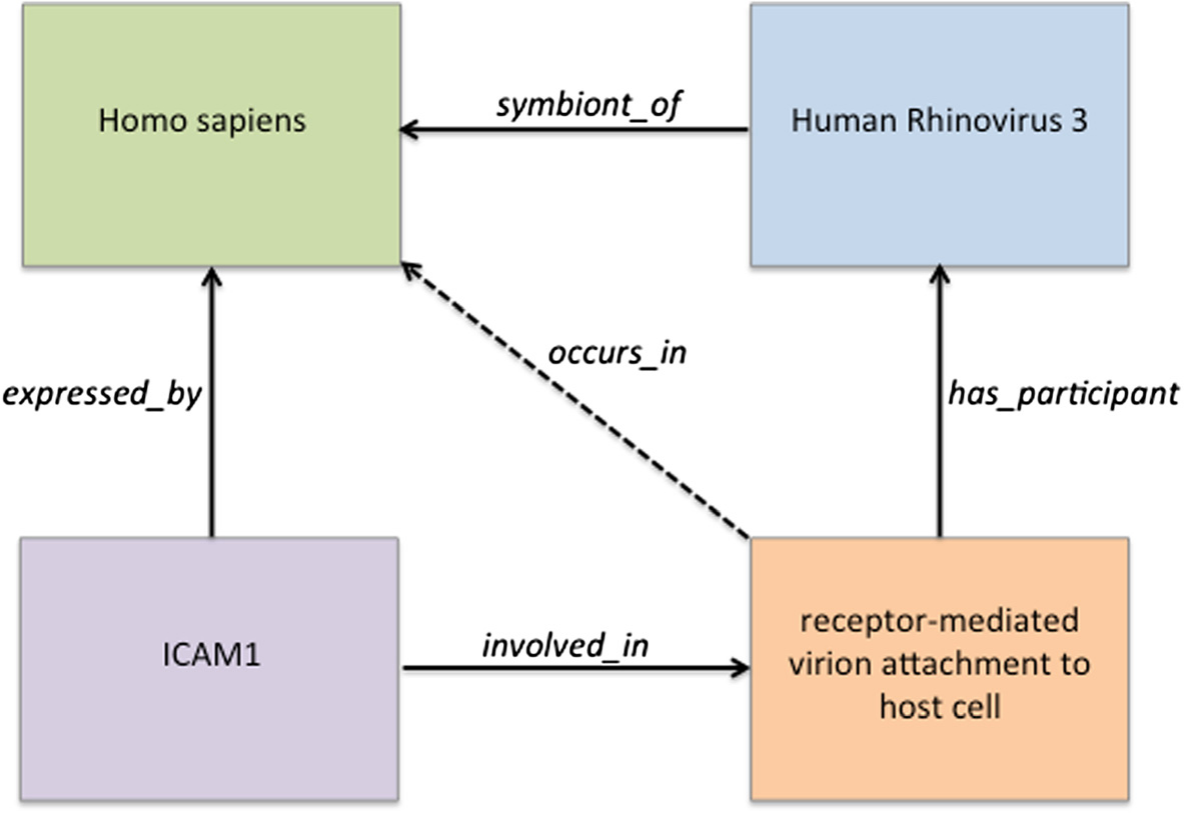
Relationships used to formalise multi-organism annotations. An extended GO annotation system will allow annotators to record relationships between interacting organisms in a multi-organism process. Here, the involvement of the host receptor, ICAM1, in receptor-mediated attachment of Human Rhinovirus 3 to human host cells is used as an example

We have extended the GO annotation system to include the means to record relationships between the organisms involved in a multi-organism process. Annotators will use the set of relations being developed in collaboration with the PCO [36] and Global Biotic Interactions (GloBI) framework [38], including ‘*symbiont_of’* , ‘*host_of’* , ‘*parasite_of’* and ‘*vector_for’* , to relate the organism expressing the annotated gene product to the other organism in the interaction. For example, ICAM1 is known to be a receptor for Human Rhinovirus 3. Previously, annotators would have simply recorded that ICAM1 is involved the process of ‘receptor-mediated virion attachment to host cell’ (GO:0046813). With the new system, they can record the interacting organism (e.g. Human Rhinovirus 3) and its relationship to the expressing organism (*symbiont_of*) (Fig. 6). Annotators can optionally record which of the two organisms the process occurs in. This is less useful for viruses, but can be important for interactions between, for example, plasmodium and its human host. The formal specification of this model uses Web Ontology Language (OWL2) [4]. This makes it easy to query the resulting annotations, for example to find all of the human genes involved in attachment of a rhinovirus to a cell.

The new relations are partly defined with reference to GO process classes that indicate types of interaction. For example, we define ‘*parasite_of*’ as a relation between two organisms (X and Y) that participate in an instance of the GO process ‘parasitism’ where X (the parasite) gains some fitness advantage from participation in the process and the interaction is disadvantageous for the fitness of Y (the host). A more complete treatment of these relations will be the subject of a forthcoming paper on the Population and Community Ontology (PCO).

### Multi-organism process GO annotations

**Fig. 7:**
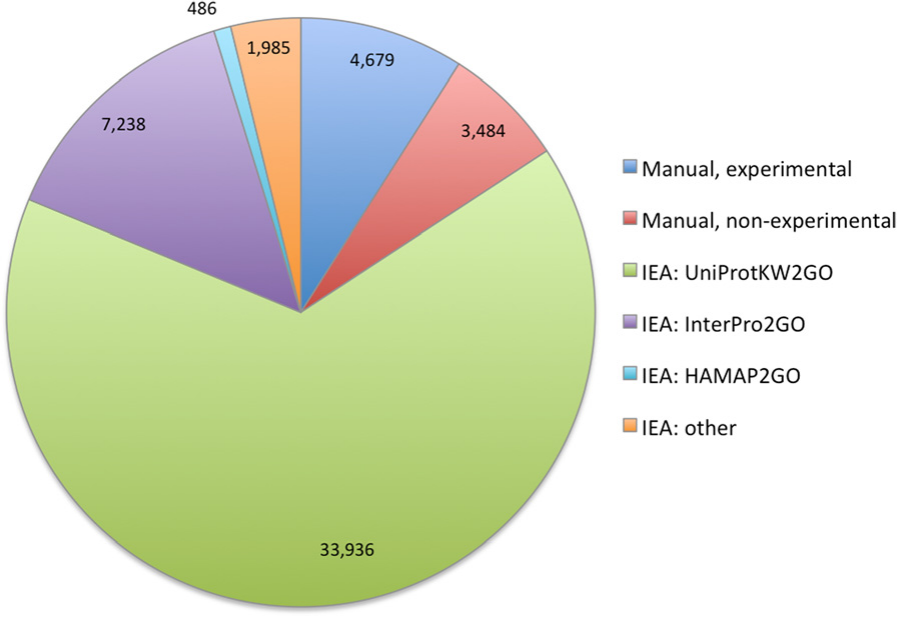
The breakdown of multi-organism process annotations into manual and electronically-derived methods. Counts exclude ‘multi-multicellular organism process’ (GO:0044706) and ‘multi-organism reproductive process’ (GO:0044703). Manual experimental annotations include annotations with evidence codes IMP, IGI, IPI, IDA, IEP and EXP. Manual non-experimental annotations include annotations with evidence codes ISS, TAS, NAS, ND, IC, RCA, IBA, IBD, IKR, IRD, ISA, ISM, ISO and IGC. Annotations with an IEA (inferred from electronic annotation) code are further broken-down into mapping methods for UniProtKB Keywords-to-GO (UniProtKW2GO), InterPro2GO, and HAMAP2GO. Refer to the text for details of the InterPro2GO and UniProtKB Keywords2GO mapping methods. For HAMAP2GO mappings, GO terms are manually assigned to each HAMAP (High-quality Automated and Manual Annotation of microbial Proteins) family rule, and transferred between highly orthologous microbial proteins. Further IEA methods are not shown for conciseness. Reactome mappings receive a TAS (traceable author statement) evidence code, and are therefore included in the manual non-experimental annotation count. Annotation counts are given for UniProtKB/Swiss-Prot identifiers which provide a non-redundant source of protein sequences, and were recorded on October 8th 2014

Existing annotations will be retrofitted to the new annotation guidelines, so information is retained for the large numbers of annotations already recorded. There are over 4 million annotations to classes in the ‘multi-organism process’ (GO:0051704) node of the GO, excluding ‘multi-multicellular organism process’ (GO:0044706) and ‘multi-organism reproductive process’ (GO:0044703) classes (October 8th 2014). Note that these latter two classes are not included in the count since these nodes mostly include classes that are not applicable to microbes such as female pregnancy and mating behavior. Given the redundancy between database sequences, it is perhaps more useful to record that over 51,000 of these annotations are associated with UniProtKB/Swiss-Prot entries. For a more detailed breakdown of the annotation count, see Fig. 7.

### Gaining annotations from mappings to external resources

Manual multi-organism annotations were provided by model organism databases and annotation groups. For viruses, UniProt focussed manual annotation efforts towards poliovirus and parvovirus B19, and EcoliWiki annotated gene products of Bacteriophage T4. To supplement the manual methods, annotations are also derived electronically from mappings of GO classes to relevant (sometimes equivalent) terms in external systems and databases. These mappings are useful both for checking consistency and for automatically deriving GO annotations. The mapping process is referenced for traceability, and most annotations assigned by this method have an ‘inferred by electronic annotation’ (IEA) evidence code. Below we describe the three key mapping methods employed for multi-organism process terms.

### Mappings between InterPro and GO

InterPro is an integrated resource of protein families [39], and InterPro entries are mapped to GO terms such that the GO function, process or component is applicable for all members of that family. This allows any new members to automatically be assigned the relevant GO term. For example, InterPro entry IPR008768 (Bacteriophage T7 capsid assembly) has a mapping to GO term ‘viral capsid assembly’ (GO:0019069). Any viral protein that contains IPR008768 will inherit an annotation to GO:0019069. As GO classes are created and modified, we work with the InterPro group to keep mappings current. Mappings are available from the GO website [40].

### Mappings between ViralZone, UniProtKB keywords and GO

Integration with the viral resource ViralZone [28] and UniProtKB keywords [18] describing host-viral processes has been crucial to the success of the revised viral GO node, and we have generated reciprocal mappings to provide interplay between all three resources; [17] describes this collaborative effort in more detail. UniProt have applied virus-host keywords to thousands of viral proteins, and GO term mappings from UniProtKB keywords are the largest source of electronic GO annotations for viral gene products (Fig. 7).

### Mappings between Reactome and GO

Reactome pathways [41] are mapped to GO terms, where possible, by Reactome curators. For example, ‘Entry of Influenza Virion into Host Cell via Endocytosis’ (REACT_6147.2) is mapped to GO term ‘receptor-mediated endocytosis of virus by host cell’ (GO:0019065) such that any Reactome protein that takes part in REACT_6147.2 receives an annotation to GO:0019065. There are two principal viral infection pathways currently annotated in Reactome; 'HIV infection' (REACT_6185.3) and 'Influenza infection' (REACT_6167.2), and one bacterial multi-organism process ‘Latent infection of Homo sapiens with Mycobacterium tuberculosis ’(REACT_121237.1). Annotations derived from these mappings are, at present, restricted to these HIV and Influenza viruses, Mycobacterium tuberculosis, and the human host.

### Taxon distribution amongst annotations

There is great taxonomic diversity across the multi-organism annotations; in UniProtKB/Swiss-Prot alone, over 4000 distinct taxons have annotations to classes in the multi-organism process node, with ‘viral process’ (GO:0016032) accounting for over half of these taxa (2179 on October 8th 2014). Table 1 lists the most-annotated taxons for viral terms in UniProtKB/Swiss-Prot, and includes both viral and host gene products.

**Table 1:** The most annotated taxons in UniProtKB/Swiss-Prot for ‘viral process’ (GO:0016032) subclasses

Organism	Taxon ID	UniProtKB/Swiss-Prot annotations per taxon
Homo sapiens	9606	1626
Mus musculus	10090	249
Influenza A virus (strain A/Puerto Rico/8/1934 H1N1)	211044	195
Vaccinia virus (strain Western Reserve)	10254	147
Human cytomegalovirus (strain Merlin)	295027	130
Vaccinia virus (strain Copenhagen)	10249	130
Human immunodeficiency virus type 1 group M subtype B (isolate HXB2)	11706	126
Human cytomegalovirus (strain AD169)	10360	125
Variola virus (isolate Human/India/Ind3/1967)	587200	114
Rattus norvegicus	10116	101
Human herpesvirus 1 (strain 17)	10299	99
Human herpesvirus 8 type P (isolate GK18)	868565	93
Epstein-Barr virus (strain B95-8)	10377	92
Human herpesvirus 2 (strain HG52)	10315	92
Human adenovirus C serotype 2	10515	82
Epstein-Barr virus (strain AG876)	82830	81
Epstein-Barr virus (strain GD1)	10376	81
Bos taurus	9913	75
Arabidopsis thaliana	3702	67
Human adenovirus C serotype 5	28285	63
Varicella-zoster virus (strain Dumas)	28285	60
Equine herpesvirus 1 (strain Ab4p)	28285	58
Human SARS coronavirus	227859	56
T4 Bacteriophage	10665	53

The annotation counts for each taxon includes manual and electronic annotation methods. The T4 bacteriophage annotation count is shown for comparison. Influenza and Human Immunodeficiency virus (HIV) are the focus of two Reactome pathways, accounting, in part, for their high annotation count. Counts taken on October 8th 2014

### Tools and software

The GO ontology structure was developed in both Web Ontology Language (OWL) and Open Biomedical Ontologies (OBO) languages using the ontology editing software OBO-Edit-2.3 [42] and Protégé 4.3 [43]. The viral GO slim was developed in OBO-Edit-2.3. We also used TermGenie [35, 44], a web-based system for template-based ontology term addition for adding new classes to GO. Gene products were manually annotated using the UniProt protein annotation tool, Protein2GO [18].

## Utility & discussion

The ontology and annotation model presented here will also allow, in the future, for the construction of complex logical queries across GO annotation data, such as “return all the host proteins involved in viral budding for Retroviridae” or “return all the symbiont proteins involved in the formation of a root nodule in soybean (*Glycine max)*”. This powerful search capability has potential uses in many areas of biological research, for example in drug discovery - “return all viral receptors for cell type X” or “return all host proteins that interact with viruses of the taxon *Paramyxoviridae*”.

The capability to make complex queries of this sort will in the future be built in to the GO tools AmiGO [45, 46] and QuickGO [12, 47], and we hope that other tool developers will leverage the information encoded into this annotation model to develop new tools and services.

### GO slim

GO ‘slims’ - subsets of the GO tailored for a particular application - have been employed widely in the analysis of large datasets e.g. [48–50]. With the volume of data arising from metagenomics and metatranscriptomics studies increasing apace, and the interest in the study of the microbiome and viriome of different environments, it is ever more important that bioinformatics tools be available to analyse these data. The GO provides both a general metagenomics slim [51] and a newly developed viral GO slim [37]. These slims are already deployed in many tools that utilize the GO, for example the EBI Metagenomics Portal [52] uses the metagenomics slim to summarize the functional profile of submitted environmental samples.

### Application of GO microbial annotation sets

Microbial GO datasets have been used to answer key questions in a wide range of biological areas including those of environmental, medical and agricultural significance. For example, in a study to examine the impact of the microbial community on the distribution of arsenic pollution in Mediterranean, Plewniak et al. [53] used GO to compare the functional composition of metagenomes from different geographical areas. The data have also been used to address medical questions such as the origin of outer membrane vesicles (OMVs) in the food-borne pathogen *Listeria monocytogenes* [54], as well as being able to examine viral protein dynamics in influenza virus H1N1 infection [55]. And in agrigenomics, GO multi-organism annotation has been used to predict drug targets for *Pseudomonas syringae* [56], an important plant pathogen that causes halo blight disease, while Peng et al. [57] used GO to help examine effectors in the potato rot nematode, *Ditylenchus destuctor*.

### Intended use of microbial multi-organism process GO terms

GO multi-species data can be used in various ways. It can be used by research scientists to interrogate large datasets, giving a functional perspective on, for example, RNASeq data. It can also be used on a gene-centric basis to provide detailed information on a particular microbe or host protein or RNA. In addition, these annotations have the potential to provide unique cross-species comparisons to allow users to identify proteins and RNA with common functions in diverse species.

By importing functional data on hosts and parasites from other resources, this project also offers the possibility of being able to consolidate data from multiple species into a single resource, thus allowing users to ask questions of a unified data set. Data from ViralZone and Reactome is already incorporated, and in the future we hope to work with the ACLAME database to align our ontologies and import their annotation data, and with the IMG resource to find ways to import their data. We will also encourage other external groups to create microbial GO annotations by providing training and advice to these groups.

As the ontology terms described in this paper are used to annotate microbial protein and RNAs, data annotators will be requesting modifications and additions to the terms, gradually improving the ontology and highlighting areas where further development is needed. We also welcome ideas for new terms, and improvements and refinements for existing terms, from members of the microbial research community. Combined with an expanding annotation set, this resource will continue to grow and improve over time.

## Conclusions

We describe here an integrated resource for providing functional data for multiple microbial species and their hosts that has applications in human and animal health, infectious disease, drug discovery, agriculture and environmental studies. In the future we will provide templates in the TermGenie tool for automated creation of these multi-organism terms, allowing users to add new terms without requiring an in-depth understanding of the ontology structure. This is a resource we expect to continue to grow and improve over time, as more groups begin to use the datasets and contribute annotations and ontology terms.

## Availability and requirements

The GO ontology classes under ‘multi-organism process’ (GO:0051704) can be viewed from any GO tool that allows visualisation of the GO, many of which require only a web-browser. To view the branch in the GO Consortium browser AmiGO 2 [45], search for ‘multi-organism process’ and then explore down through the tree via the ‘inferred tree view’ or ‘ancestors and children’ tabs. Annotated genes and proteins can be viewed at any level in the tree by selecting the ‘Associations’ tab. The multi-organism process ontology and annotated proteins can also be viewed and downloaded from the UniProt GO browser QuickGO [12]. This is done by searching for ‘multi-organism process’ and navigating down the tree using the ‘Child terms’ tab. Annotated proteins for the selected term can be viewed in the ‘Protein Annotation’ tab. The annotated proteins can be further filtered e.g. by taxon using the ‘Filter’ option and downloaded with the ‘Download’ button.

The multi-organism process ontology can be downloaded as a part of the GO ontology resources in OWL or OBO format [58].

## Abbreviations

CARO: Common Anatomy Reference Ontology
EFO: Experimental Factor Ontology
GO: Gene Ontology
HAMAP: High-quality Automated and Manual Annotation of microbial Proteins
IDO: Infectious Disease Ontology
IMG: Integrated Microbial Genome Network
OBI: Ontology for Biomedical Investigations
PAMGO: Plant-Associated Microbe Gene Ontology consortium
PCO: Population and Community Ontology
UniProtKB: UniProt KnowledgeBase

## Footnotes

1 We specify this in OWL as: ‘membrane fusion’ *that* has_participant *min* 2 ‘organism, virus or viroid’. Note that the PCO [36] and CARO (Common Anatomy Reference Ontology) [59] projects plan to integrate the OBI ‘organism’ term [33].
